# Exploring the mechanistic pathways of how social network influences social norms in adolescent smoking prevention interventions

**DOI:** 10.1038/s41598-023-28161-7

**Published:** 2023-02-21

**Authors:** Felipe Montes, Martha Blanco, Andres F. Useche, Sharon Sanchez-Franco, Carlos Caro, Lei Tong, Jie Li, Huiyu Zhou, Jennifer M. Murray, Olga L. Sarmiento, Frank Kee, Ruth F. Hunter

**Affiliations:** 1 Department of Industrial Engineering, Social and Health Complexity Center, Universidad de Los Andes, Bogotá, Colombia; 2 Department of Public Health, School of Medicine, Universidad de Los Andes, Bogotá, Colombia; 3 School of Informatics, University of Leicester, Leicester, UK; 4 College of Electronic and Information Engineering, Nanjing University of Aeronautics and Astronautics, Nanjing, China; 5 Centre for Public Health, Institute of Clinical Sciences, Block B, Queen’s University Belfast, Royal Victoria Hospital, Grosvenor Road, Belfast, BT12 6BA, 02890978955 UK; 6 Centre for Public Health, Institute of Health Sciences, School of Medicine, Dentistry and Biomedical Sciences, Queen’s University Belfast, Belfast, BT12 6BJ UK

**Keywords:** Disease prevention, Risk factors, Complex networks

## Abstract

We know little about how smoking prevention interventions might leverage social network structures to enhance protective social norms. In this study we combined statistical and network science methods to explore how social networks influence social norms related to adolescent smoking in school-specific settings in Northern Ireland and Colombia. Pupils (12–15 years old) participated in two smoking prevention interventions in both countries (n = 1344). A Latent Transition Analysis identified three groups characterized by descriptive and injunctive norms towards smoking. We employed a Separable Temporal Random Graph Model to analyze homophily in social norms and conducted a descriptive analysis of the changes in the students’ and their friends’ social norms over time to account for social influence. The results showed that students were more likely to be friends with others who had social norms against smoking. However, students with social norms favorable towards smoking had more friends with similar views than the students with perceived norms against smoking, underlining the importance of network thresholds. Our results support the notation that the ASSIST intervention takes advantage of friendship networks to leverage greater change in the students' smoking social norms than the Dead Cool intervention, reiterating that social norms are subject to social influence.

## Introduction

Globally, around 43.8 million adolescents aged 13–15 years old smoke cigarettes or use smokeless tobacco products^1^. Further, 90% of adult smokers typically start smoking as a teenager (< 19 years old)^2^ and the tobacco industry has strategically developed new products especially attractive to young people^3^. Among adolescents, peer influence over smoking behavior is strongest^4–6^, therefore, this is a critical life stage in the prevention of tobacco consumption.

Behavior change strategies have been useful for reducing health risks among adolescents^7–9^. Some behavior change theories argue that individuals' emotions, attitudes, and behaviors can exert interdependent effects on the behaviors of others^10^. However, several studies have demonstrated that social norms can influence a broad range of health behaviors and behavioral changes during public health interventions^10,11^.

Social norms are the standard expectations of a community regarding what they believe to be a typical and appropriate action^12^. Beliefs about what others do are *descriptive* social norms, and beliefs about what others think one *should* do are *injunctive* social norms^10,11^. Furthermore, social norms depend on context and social networks and operate differently for different behaviors^13^. Moreover, changes in social norms will influence individuals' behaviors and decisions^14,15^. Indeed, Murray et al.^16^ offered evidence that high-status peers may influence levels of susceptibility to risky behavior such as smoking during adolescence.

There is a growing interest in understanding how we can best use social networks in health behavior change interventions^17–19^. These changes may depend on network processes like homophily and peer influence. Homophily, also referred to as homophily selection, is the principle by which similar behaviors and attributes can cause interpersonal attraction and increases the contact between people^20,21^. In contrast, influence is a process in which an ego assimilates to the behavior of peers and changes his or her own behavior^21^. Peer influence refers to a process affecting a person’s behavior that is generated from an overt or subliminal comparison with the behavior of other people with whom that person is connected^22^. Previous studies have shown that adolescents’ school friends, and their smoking behaviors, exert peer influence on their tendency to smoke^23,24^. Although the importance of social networks and peer influences on health behaviors among adolescents is well established [e.g.^19^], there is limited evidence on how they influence normative behaviors or the mechanisms which underlie social norms-based interventions in different contexts.

Smoking prevention interventions among adolescents leverage peer influence as an important behavior change process^7^. The Mechanisms of Networks and Norms Influence on Smoking in Schools (MECHANISMS) study^25^ used two school-based interventions that aim to prevent adolescent smoking but operate via contrasting theoretical frameworks and mechanistic pathways. The A Stop Smoking in Schools Trial (ASSIST) leverages social networks and social influence processes to train students to maintain preventive social norms about tobacco and smoking^26^. By contrast, Dead Cool is a teacher-led intervention where students are taught skills to identify and manage social influences for tobacco use using didactic lessons^27^. The ASSIST intervention uses peer influence to facilitate changes emerging from social interactions while the Dead Cool intervention uses a direct classroom approach where the teacher is the deliverer of the message. The interventions targeted students aged 12–15 years old, for whom actual smoking is typically rare, and aimed to maintain the norm of non-smoking. Each intervention was implemented in three schools in Northern Ireland (NI) and three schools in Bogotá, Colombia (COL), for a total of 12 schools. There are differences between smoking rates between high-income countries (such as NI) and low-middle income countries (such as Colombia). This study takes into account these cultural differences to analyze the two smoking prevention interventions^16,25^.

This study was undertaken as part of the MECHANISMS project that seeks to contrast peer-based and teacher-led school-based smoking prevention interventions among adolescents in the UK and Colombia^7^. This study is helpful for understanding the importance of the social network structure in the dynamics of social norms. The aim of this study was to investigate if homophily or social influence are present as mechanistic pathways by which social network structures influence social norms before and after both interventions. We also compare the results between interventions in COL and NI schools to understand if they are driven by different contexts. For the analysis, first, we identified the change in social norms by classifying the students into latent groups associated with perceived social norms before and after the interventions. Second, we evaluated changes in the friendship network structures over time. Finally, we explored if the change in the social network structure is related to transitions across the social norms groups and the potential role of homophily or social influence in this evolution during the interventions. To our knowledge, this is the first study to explore social network structure processes (i.e. influence and homophily) on experimentally derived social norms^16,28^.

## Results

In this section, first, we characterize students' change in social norms by classifying them according to their social norms before and after the intervention. Next, we describe the change in students' friendships as the network changes over time. Finally, we explored the relationship between the changes in the social norms groups and the changes in the friendship network to assess the presence of homophily or influence.

### Uncovering the latent groups related to social norms

Seeking to describe the change in the smoking social norms, we classified the students into latent groups associated with smoking social norms before and after the smoking prevention interventions. Grouping students according to descriptive and injunctive smoking social norms is an objective way of obtaining attributes for each individual according to the label of the group to which they belong. In addition, having a clear distinction of latent group change avoids ambiguity in the measurement of change in social norms. The groups emerged from the data and were labeled according to *descriptive* and *injunctive* social norms favorable towards smoking or against smoking. We conducted an initial classification with a Component-based Feature Saliency for Clustering (CFSC)^29^ and followed this with a Latent Transition Analysis (LTA) to understand the transitions of participants between groups over time (see “Methods”)^30,31^. The LTA identified three latent groups (Table 1) for each time-point (baseline and follow-up) according to the Bayesian information criterion (BIC) for dissolution that was 0.92, indicating a high certainty in the classification.

**Table 1:** The proportion of participants aggregated for both settings belonging to each latent group by intervention according to the Latent Transition Analysis.

Social norms latent group	ASSIST		Dead cool	
	Baseline	Follow-up	Baseline	Follow-up
Descriptive social norms favorable towards smoking	15%	13%	7%	8%
Both social norms against smoking	82%	85%	91%	89%
Injunctive social norms favorable towards smoking	3%	2%	2%	3%
Total	100%	100%	100%	100%

The first latent group included 116 students (11.4%) at baseline and 109 (10.7%) students at follow-up. This group was characterized by students with *descriptive* social norms favorable towards smoking and *injunctive* social norms against smoking. This suggests that students in this group believe that other people around them smoke, but they believe that people around them believe that smoking is not socially acceptable. The second latent group included 878 students (86.2%) at baseline and 885 (86.9%) at follow-up. This group was characterized by students with *descriptive* and *injunctive* social norms against smoking. This suggests that students in this group believe that other people around them do not smoke and that they believe that smoking is not socially acceptable. Finally, the third latent group included 24 students (2.4%) at baseline and 24 (2.4%) at follow-up. This group was characterized by students with *injunctive* social norms more favorable towards smoking. This suggests that students in this group perceive social acceptance toward smoking, regardless of whether they believe that people around them smoke or not. There was similar group membership irrespective of the intervention being received by the students.

**Figure 1:**
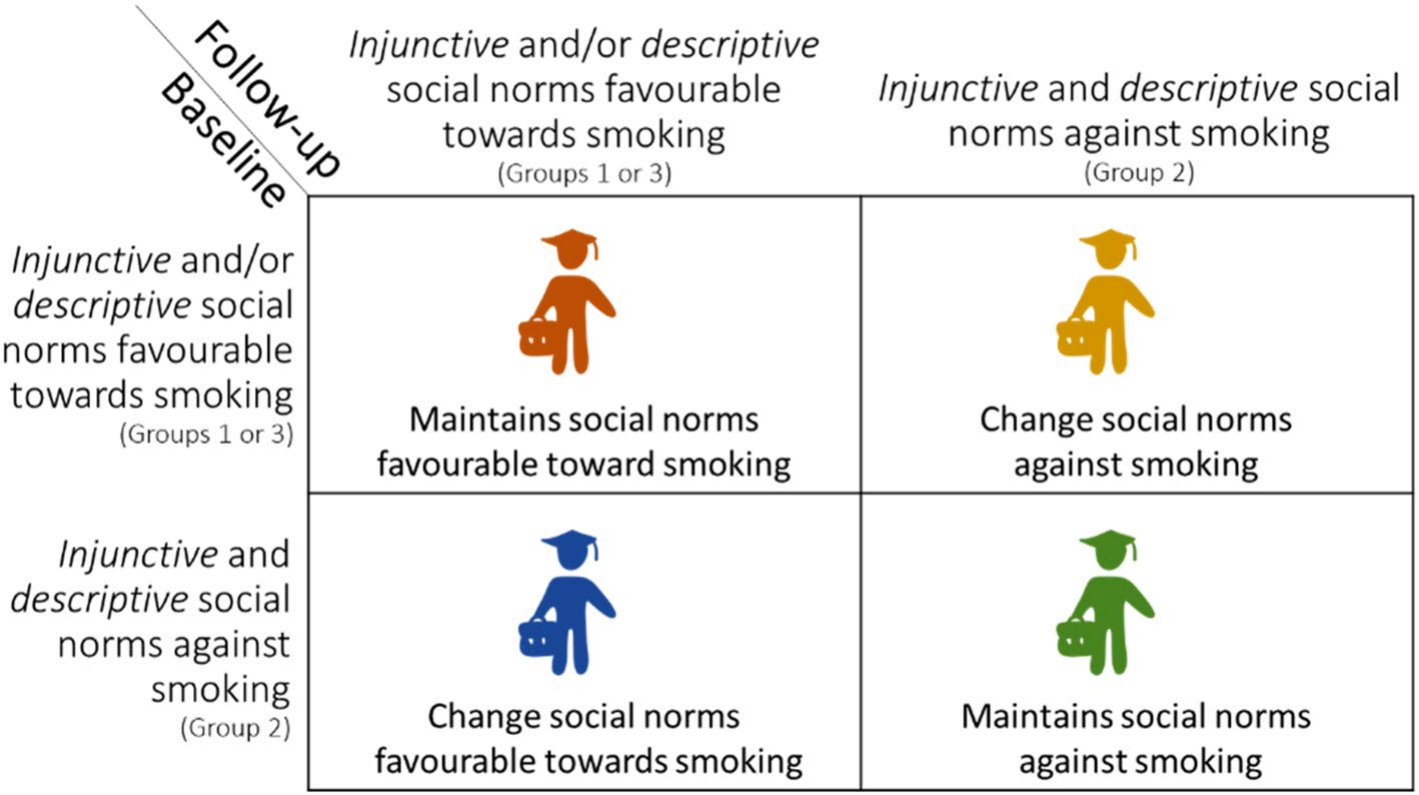
Possible changes in the student social norms from baseline to follow-up: change social norms to favorable towards smoking, change social norms to against towards smoking, maintain social norms favorable towards smoking, or maintain social norms against smoking.

According to the LTA results, transitions (Fig. 1) reflected a change in the students who had social norms favorable towards smoking, in general. In fact, 55 students changed their social norms from being *favorable* towards smoking to being *against* smoking after the intervention (50 students representing 43% of the students at baseline in the group with *descriptive* social norms favorable towards smoking, and 5 students representing 21% of the students at baseline in the group with *injunctive* social norms favorable towards smoking). In contrast, 48 students changed their social norms to be *favorable* towards smoking after the intervention (5% of students who were in the group with *injunctive* and *descriptive* social norms against smoking at baseline).

We conducted a chi-square test at the 5% level of significance to assess whether transitions between the groups with social norms toward smoking and the group with both social norms against smoking are independent of the type of intervention. We observed that for both settings the students who received the ASSIST intervention, which is based on peer influence and purposefully utilizes the social network structure for intervention implementation, showed a greater group transition to having both social norms *against* smoking than students who received the Dead Cool intervention (coefficient of 3.1). Of the total number of students who switched to a social norms group to against smoking in NI, approximately 86% participated in the ASSIST intervention, while among those who made this transition in COL, 59% participated in the ASSIST intervention. This suggests a positive effect on both settings, with a greater impact in NI.

Finally, we conducted a multinomial logistic regression (Table 2) to characterize the latent groups at baseline according to self-reported variables including socio-demographic variables (age, sex, home composition, ethnic status), psychosocial traits like self-efficacy, pro-sociality (need to belong, fear of negative valuation, pro-sociality), and personality (openness, extraversion, agreeableness, conscientiousness, stability)^25^. This analysis allows us to characterize and validate the students in these latent (social norms) groups, while in addition these same variables will also be analyzed in the evaluation of homophily. We found that age, sex, and setting were associated with group assignment (p-value < 0.05). **Older students** were more likely to be assigned to a group with *injunctive* and/or *descriptive* social norms favorable toward smoking than to the group with both social norms against smoking, in comparison to younger students (11 years old, Table 2). **Girls** were more likely than boys to be assigned to the group with descriptive social norms favorable towards smoking than to the group with both social norms against smoking (odds ratio [OR]: 1.70, confidence interval [CI]: [1.05;2.74]). This suggested that girls were more likely to be in a group with *descriptive* social norms favorable towards smoking than boys. Students from NI were more likely than students from COL to be assigned to the group with descriptive social norms favorable towards smoking over the group with both social norms against smoking (OR:1.88, CI: [1.06;3.31]). This suggested that students from NI were more likely to be in a group with *descriptive* social norms favorable toward smoking. There were no significant group associations with *home composition* or *ethnic status* variables.

**Table 2:** Characterization of each latent group at baseline obtained in the LTA according to the intervention, sociodemographic and psychosocial traits conducting a multinomial statistical regression odds ratio estimates.

Variable type	Variable	Group with descriptive social norms favorable towards smoking	Group with injunctive social norms favorable towards smoking
–	Intercept (A)	9.61E−10***	4.34E−5***
Intervention	Dead cool	0.76	0.27*
Sociodemographic	Sex = Girl	1.70*	1.00
	Sex = Prefer not to say	0.55	9.12E-17
	Age = 12 years	1.74E10***	2.25E4***
	Age = 13 years	2.72E10***	2.43E4***
	Age = 14 years	3.42E10***	2.12E4***
	Age = 15 years or more	4.04E10***	2.19E4***
	Ethnic minority = No	1.10	2.06
	Home composition = both parents	1.00	0.81
	Home composition = other adults	1.23	2.31
	Setting = NI	1.88*	1.13
Psychosocial traits	Self-efficacy	0.49***	1.27
	Need to Belong	1.11	0.54
	Fear of Negative Evaluation	0.96	1.52
	Prosocial Behavior	0.89	1.06
	Openness (Big Five’s subscale)	1.08	0.72
	Extraversion (Big Five’s subscale)	1.59*	1.18
	Agreeableness (Big Five’s subscale)	0.67	0.35*
	Conscientiousness (Big Five’s subscale)	0.59*	0.40
	Emotional stability (Big Five’s subscale)	0.78	0.79

The reference categories for each variable are as follows: group with both social norms against smoking for latent groups, boy for sex, 11 years for age, yes for ethnic minority, “single parent” for home composition, COL for setting, and ASSIST for intervention. *< 0.05 **< 0.01 ***< 0.001,

Regarding the students’ psychological characteristics, we found that self-efficacy and three of the five personality traits (extraversion, agreeableness, and conscientiousness) were associated with group assignment, with a p-value < 0.05 (Table 2). For an increase of 0.1 on the **self-efficacy** scale, students were less likely to be assigned to the group with *descriptive* social norms favorable towards smoking than to the group with *descriptive* and *injunctive* social norms against smoking (, OR: 0.49, CI: [0.37;0.65]). For an increase on the **extraversion** scale, students were more likely to be assigned to the group with *descriptive* social norms favorable toward smoking than to the group with *descriptive* and *injunctive* social norms against smoking (OR: 1.59, CI: [1.11;2.28]). For an increase on the **agreeableness** scale, students were less likely to be assigned to the group with *injunctive* social norms favorable toward smoking than to the group with *descriptive* and *injunctive* social norms against smoking (OR: 0.67, CI: [0.42;1.05]). For an increase on the **conscientiousness** scale, students were less likely to be assigned to the group with *descriptive* social norms favorable toward smoking than to the group with *descriptive* and *injunctive* social norms against smoking (OR: 0.59, CI: [0.38;0.94]). Pro-sociality characteristics (need to belong, fear of negative evaluation, pro-sociality) and two of the five personality trials (openness and emotional stability) were not significantly associated with group assignment.

### Assessing the change in network structure over time

**Figure 2:**
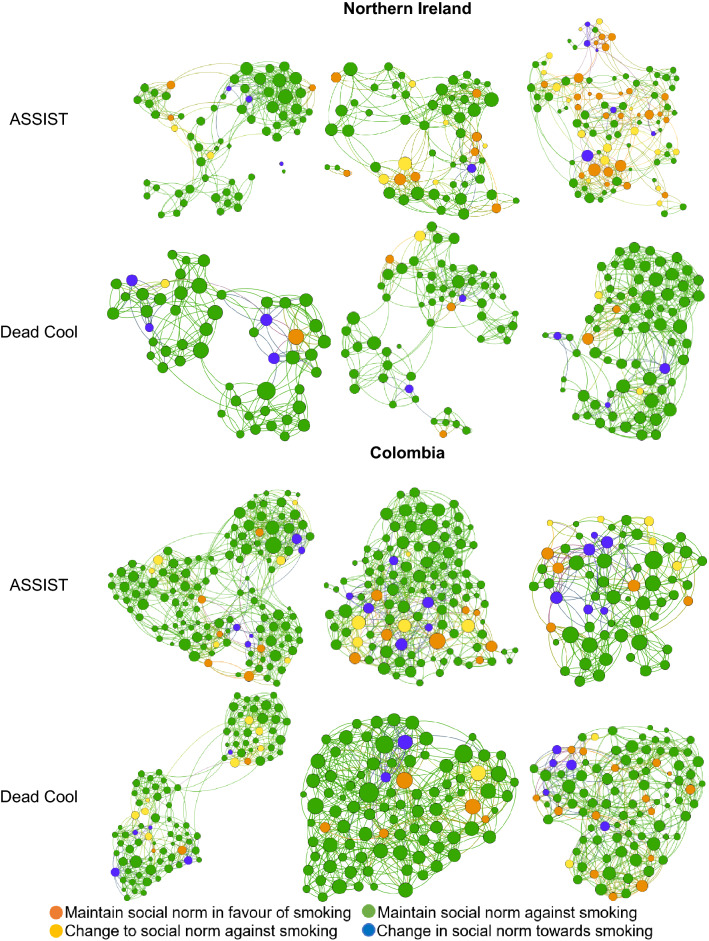
Friendship networks, sorted by setting and intervention. Nodes: students. Edges: friendship nominations at baseline. For nodes: color is the corresponding change in social norm, size is the in-degree.

Second, we explored whether the friendship network changed before and after the intervention to discuss the relevance of studying the relationship between social norm change and friendship change. We assessed 12 directed networks, two per school, one at baseline and the other at follow-up (Fig. 2). Networks were defined by the friendship nomination (ties) between the students (nodes). Social networks were similar in size across schools and settings over time (Table 3). Of the 12 school networks, the number of nominated friendship nodes decreased on average by 1.75% from baseline to follow-up (nodes ranged from 83 to 177 at baseline and from 77 to 176 at follow-up), while the average *in-degree*, representing the number of received friendship nominations per node, remained at 6 friends on average after the intervention.

**Table 3:** Friendship networks measures.

Schools	Time point	Nodes	Ties	Average in degree	Density	Jaccard index
NI-dead cool	Baseline	83	537	6.5	0.08	0.44
	Follow-up	82	524	6.4	0.08	
	Baseline	111	851	7.7	0.07	0.46
	Follow-up	112	756	6.8	0.06	
	Baseline	97	706	7.3	0.08	0.56
	Follow-up	97	787	8.1	0.08	
NI-ASSIST	Baseline	119	849	7.1	0.06	0.40
	Follow-up	117	755	6.5	0.06	
	Baseline	122	787	6.5	0.05	0.40
	Follow-up	121	740	6.1	0.05	
	Baseline	177	1230	6.9	0.04	0.42
	Follow-up	176	1015	5.8	0.03	
COL-dead cool	Baseline	106	703	6.6	0.06	0.42
	Follow-up	103	651	6.3	0.06	
	Baseline	99	743	7.5	0.08	0.41
	Follow-up	99	641	6.5	0.07	
	Baseline	126	739	5.9	0.05	0.38
	Follow-up	121	590	4.9	0.04	
COL-ASSIST	Baseline	144	957	6.6	0.05	0.34
	Follow-up	141	796	5.6	0.04	
	Baseline	126	828	6.6	0.05	0.33
	Follow-up	124	775	6.3	0.05	
	Baseline	84	473	5.6	0.07	0.26
	Follow-up	77	374	4.9	0.06	

Nodes represent individual participants; Ties represent the friendship nomination between participants; average in degree represents the mean number of friends nominated per individual participant; density is the ratio of the number of ties observed to the number of possible ties for a given network. Jaccard Index measures the structural change between follow-up and baseline, thus there is only one coefficient per school over time.

Then, we measured the Jaccard index to assess changes in the network structure over time. The Jaccard index measures the amount of change in the ties over time within the network and was used for its simplicity in handling continuous and categorical variables. The resulting values ranged from 0.26 to 0.56. (Table 3). All but one school was found to have significant changes in the structure of friendship ties over time.

### Assessing the relationship between the network structure and the social norms latent groups

Third, given that the networks showed structural changes over time, we analyzed homophily and social influence mechanisms related to changes in the latent groups of social norms and the changes in the network. We implemented a Separable Temporal Exponential Random Graph Model (STERGM) to analyze homophily according to social norms and used descriptive analysis to analyze social influence among participants.

#### Formation and dissolution of ties related to social norms (homophily effects)

The STERGMs estimate the likelihood of the formation and dissolution of ties related to social variables over time^32^. By applying the STERGM, overall, we found that the formation and dissolution of friendships had no significant association with being assigned to any particular social norms group. This implies that there is little homophily around smoking-related social norms among students. In the STERGM model outcomes, we observed that transitivity (i.e. that friends of friends are also friends) and reciprocity (i.e. that a friendship nomination is returned) measures were significant and positive concerning the formation and dissolution of friendship ties in the 12 networks (Table 4). The transitivity results suggest that students were more likely to form friendships with students who had mutual friends and to preserve friendships when there were mutual friends. The reciprocity results tell us that students were more likely to form and preserve reciprocal friendships. Social norms groups from the LTA at baseline had a significant bearing on the formation of friendship ties in 3 of the 12 networks, and social norms group at follow-up had a significant effect on the dissolution of friendship ties in 2 of the 12 networks (Table 4).

**Table 4:** Coefficients and p-values resulting from the STERGM model for each network.

School		Formation			Dissolution		
		Reciprocity	Transitivity	Social norms group on baseline	Reciprocity	Transitivity	Social norms group on follow-up
NI- Dead Cool	Estimate	3.09	3.00	− 0.15	1.01	0.59	− 0.11
	p-Value	0.00***	0.00***	0.27	0.00***	0.00***	0.52
	Estimate	3.64	3.32	0.02	0.93	0.70	0.10
	p-Value	0.00***	0.00***	0.89	0.00***	0.00***	0.46
	Estimate	3.77	4.48	0.04	0.81	0.83	− 0.08
	p-Value	0.00***	0.00***	0.73	0.00***	0.00***	0.66
NI- ASSIST	Estimate	3.43	2.54	0.19	1.13	0.72	0.27
	p-Value	0.00***	0.00***	0.10*	0.00***	0.00***	0.04**
	Estimate	2.77	3.84	0.00	0.83	0.78	0.02
	p-Value	0.00***	0.00***	1.00	0.00***	0.00***	0.90
	Estimate	3.38	3.74	0.08	0.66	0.79	0.21
	p-Value	0.00***	0.00***	0.46	0.00***	0.00***	0.07*
COL- Dead Cool	Estimate	3.88	5.08	− 0.37	1.22	0.59	0.04
	p-Value	0.00***	0.00***	0.00***	0.00***	0.00***	0.79
	Estimate	3.06	5.25	0.41	1.67	0.52	− 0.11
	p-Value	0.00***	0.00***	0.03**	0.00***	0.00***	0.51
	Estimate	3.66	4.63	0.14	1.18	0.68	N/A
	p-Value	0.0001***	0.0001***	0.386	0.0001***	0.0001***	N/A
COL- ASSIST	Estimate	3.82	4.33	0.06	1.60	0.85	-0.13
	p-Value	0.00***	0.00***	0.65	0.00***	0.00***	0.33
	Estimate	3.73	4.92	0.07	1.60	0.69	N/A
	p-Value	0.0001***	0.0001***	0.51	0.0001***	0.0001	N/A
	Estimate	3.83	6.11	− 0.13	1.06	0.85	0.11
	p-Value	0.00***	0.00***	0.42	0.00***	0.00***	0.54

Transitivity represents the probability that friends of friends are also friends; Reciprocity represents the percentage of friendship nomination that is returned; N/A did not converge; p < 0.1; *p < 0.05; ***p < 0.00.

#### Changes in social norms associated with peer influence (social influence effects)

**Figure 3:**
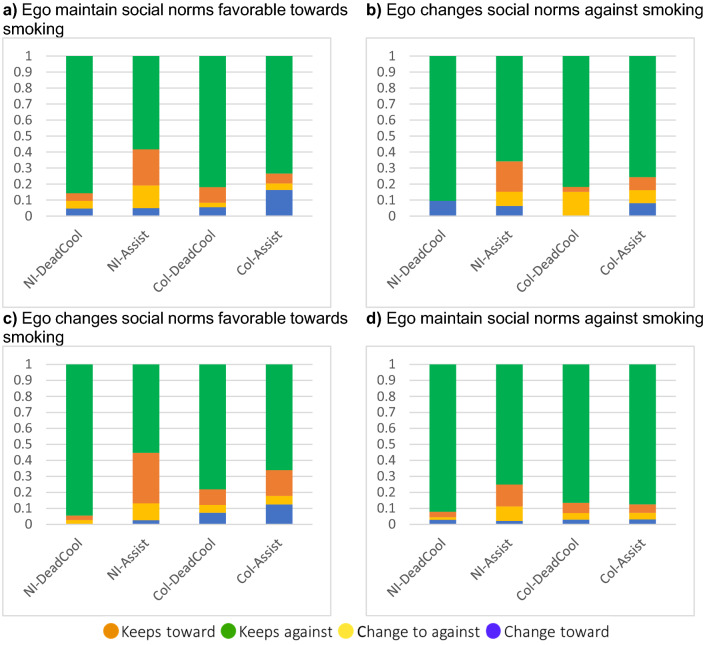
The proportion of friends that were maintained from baseline to follow-up by an ego (i.e. the individual participant) that changed their social norms, according to the change in the social norms of the ego. Each caption represents the proportion of ego’s friends that changed their social norms, differentiating by setting and intervention, according to the change of the ego’s social norms. Green represents students who maintained social norms against smoking, orange represents students who maintained social norms favorable towards smoking, yellow represents students who changed social norms to against smoking, and blue represents students who changed social norms to favorable towards smoking. For example, (**a**) shows the proportion of friends that changed or maintained their social norms for the egos that maintained their social norms favorable towards smoking.

We analyzed the change in the students’ social norms group over time, according to the group changes of their nominated friends. The analyzed data are shown in Fig. 3. We described the group changes for friends who remained friends at *both time* points (n = 3579 ties at baseline and follow-up), the newly nominated friends at *follow-up* (n = 2029 ties only at follow-up), and the friends that were nominated only at *baseline* but not at follow-up (n = 2392 ties only at baseline). We separately assessed the students according to their social norms changes. Depending on their own change (i.e. ego), we measured the average percentage of friends who changed or maintained their social norms group (Fig. 3). This analysis was carried out differentiating by setting (NI or COL) and intervention (represented by bars in the graphs of Fig. 3).

Regardless of the change in the students' own social norms, most of their friends changed towards or remained in, the group of social norms against smoking. In fact, the average proportion of friends with *injunctive* and *descriptive* social norms against smoking at follow-up ranged between 55 and 94% (average 78%) for those who were friends at *both time points*, 64% and 100% (average 80%) for those who were friends only at *follow-up*, and 50% and 100% (average 100%) for those who were friends only at *baseline*. This suggested that students are more likely to nominate more friends who remained in the social norms group against smoking, regardless of their social norms change, intervention, and setting.

Finally, we measured the percentage of friends at baseline that had social norms towards and against smoking, for each possible scenario of social norms change. In respect of the students who maintained or changed their social norms to be favorable towards smoking, the average percentage of friends with norms that were favorable towards smoking at baseline was 22% and 21% respectively. In contrast, in respect of the students who maintained or changed their social norms to be against smoking, the percentage of friends with norms that were favorable towards smoking at baseline was 12% and 19%, respectively. We did not find differences by setting or intervention. This suggested that students who maintained or changed their social norms to be in favor of smoking were likely to have more friends in favor of smoking at baseline than the students who sustained or changed their perceived social norms to be against smoking, regardless of their setting and intervention.

## Discussion

We explored how adolescents’ social network structures, defined by friendship nominations, influence smoking social norms in the context of two smoking prevention interventions implemented in six schools in Bogotá (Colombia), and six schools in Northern Ireland (United Kingdom) using MECHANISMS study data^25^. We identified three latent groups of participants classified according to *descriptive* and *injunctive* social norms favorable towards or against smoking and analyzed the change of group membership together with social network analysis to assess the role of influence and homophily regarding smoking social norms in the context of two interventions and in the two countries. To our knowledge, this is the first study to explore the influence of social network structures on experimentally derived social norms based on game theory experiments yielding measures less susceptible to social desirability bias^33^.

We identified three latent groups associated with descriptive and injunctive social norms around smoking. The first group included students with *descriptive* social norms favorable towards smoking and *injunctive* social norms against smoking. The second group included students with *descriptive* and *injunctive* social norms against smoking. The third, and by far the smallest group, included students with *injunctive* social norms favorable towards smoking (irrespective of their perceived *descriptive* norms). These findings further illustrate the potential of LTA to identify latent groups of social norms for smoking in different contexts. This study is novel in that it uses LTA to identify groups related to smoking social norms in the context of prevention interventions rather than for population already smoking. Other studies have used LTA to identify groups related to smoking exposure and smoking status^34^, transitions through the stages of change for smoking cessation^35^, with other studies related to smoking in adolescents and young adults aiming to distinguish subgroups with different smoking prevalence^36^ and to characterize diverse groups according to tobacco and nicotine product use^37^.

We found that slightly older students were more likely to belong to a group with *injunctive* and *descriptive* social norms favorable towards smoking. This result is similar to previous studies that have shown that susceptibility towards smoking and smoking rates increase rapidly with age^38^. Furthermore, we found that girls were more likely to be assigned to a group with descriptive social norms favorable towards smoking than boys^39^. Women are a new target population of tobacco marketing, in which smoking is offered as a symbol of empowerment^39^. This can impact their perception of the cigarette and, therefore, their social norms. We found that self-efficacy, agreeableness, and conscientiousness were inversely associated with the propensity to belong to a group with social norms *favorable* toward smoking, while extraversion was positively associated. Our results are coherent with previous evidence showing that improvements in self-efficacy are related to reductions in smoking susceptibility^40^, and suggest that higher levels of self-efficacy can reduce the probability of belonging to a group with *descriptive* social norms favorable towards smoking. Our results were inconclusive regarding the relationship between pro-sociality traits (need to belong, fear of negative evaluation, and pro-sociality) and belonging to any particular smoking social norms groups, even though other studies have shown a negative relationship between pro-sociality traits and smoking susceptibility^41,42^ and a negative relation between pro-social influence and age^43^.

Regarding homophily (i.e. friendships between people who have attributes in common), friendship ties are formed and maintained in association with common and reciprocal friendships, but we did not find changes in friendship ties related to smoking norms groups. This result is consistent with the discussion by Valente and Pitts^44^, where natural network evolution is purported to connect friends in common by unobservable variables. The authors named this phenomenon 'latent homophily', and it describes how students may be connected for unobserved reasons in a study (e.g. behaviors or activities outside the school, like physical activity, specific sports, or participating in music groups). This result suggests that belonging to the identified social norm groups does not drive homophily in this sample of students, however, other characteristics may incentivize homophily among school students like having reciprocal friendship relationships.

After the ASSIST and Dead Cool school-based interventions, most students remained or transitioned to the group with social norms against smoking though approximately 5% of the students transitioned to a group with norms favorable towards smoking. We found differences in transitions between groups according to the smoking prevention intervention delivered in the schools. Regarding the influence process (i.e. change in the person’s behavior generated by her or his friendships) in the schools, we found that the ASSIST intervention seems to promote more transitions to groups with social norms against smoking than the Dead Cool intervention. This outcome reflects the intended underlying mechanism of the ASSIST intervention, which was intentionally designed to use the social network structure of peers to diffuse anti-smoking norms, while Dead Cool is based upon traditional classroom pedagogy. Our results support the idea that social norms are subject to a type of social contagion within social networks^45^. This finding illustrates the potential of social influence acting in friendship networks to contribute to smoking prevention, even in culturally different networks^46^. However, further research is needed to understand the optimal network intervention strategy to influence social norms. In fact, there are many different types of network intervention approaches^17,18,47–49^ and ASSIST uses only one type of network targeting approach (i.e. nominated influential students from the school year)^25,50^. Also, we found that students who retain or change to social norms *favorable* towards smoking are likely to have more friends with similar views at baseline than the students with perceived norms against smoking. This result is observable regardless of their setting and intervention. This finding underlines the importance of network thresholds to behavior adoption and change^44,47–49^. Network thresholds are represented by the proportion of connections to friends in the personal network that have a behavior before the ego adopts it^44^.

Our findings should be interpreted in the light of the following limitations. First, 1407 students were analyzed in the network, 1394 at baseline and 1370 at follow-up, but only 1018 students had complete data on social norms and sociodemographic attributes for both time points. However, the analyzed network data corresponds to 72% of the total students and this subgraph preserves a similar in-degree distribution as that of the entire network, according to a t-test conducted among both degree distributions. This means that the analyzed subsample preserves a similar network structure. Despite the reduction in the sample, the sub-sample remains diverse in terms of sociodemographic attributes, thus our results permit us to make robust inferences about differences in transitions based on age, gender, setting, and intervention. Second, there is no control group to assess the effect of the interventions in terms of changes in the friendship network structure and changes in the social norms—the study was not designed to test intervention effectiveness, but to provide a detailed exploration of mechanistic pathways. Nevertheless, we can compare the effect of peer influence and homophily among students offered the ASSIST and Dead Cool interventions according to their different designs. This is a relevant outcome for the MECHANISMS study, which was not designed to offer head-to-head comparisons of the effectiveness of the interventions, which have both been previously tested, but was designed to compare and contrast their intervention mechanisms^25^. Third, even if we were able to measure some changes in social norms and the network structures during our short study period, a longer period of implementation and observation of the programs might be needed to observe prominent changes attributable to peer influence and homophily effects, and social norms. Finally, social desirability bias can be affected by personal^51^ and social network characteristics^52^ and can affect the way that norms are reported^51,52^. This in turn could obscure the identification of social network effects *on* social influence and even the nature *of* the social influence effects that are occurring. This study is unique in distinguishing and accounting for both self-report social norms and experimentally derived incentivized social norms. Previously, Murray et al.^16^ showed evidence for the construct validity of eliciting adolescent smoking norms through self-report and experimentally derived incentivized methods. Furthermore, it is important to identify social norms separately from behavior in a longitudinal analysis that can distinguish the determinants of change in each^16,28^.

In conclusion, the ASSIST intervention, which is designed to leverage peer influence in school friendship networks, can help prevent adolescent smoking uptake by increasing the proportion of students that change to social norms against smoking. This effect was higher for students receiving ASSIST than for the Dead Cool intervention, which does not purposefully leverage friendship networks or personal attributes. Intervention design features that are important for network-based interventions include the threshold proportion of friends at baseline with social norms favorable towards smoking. This suggests the presence of an influence process to change social norms, determined by the type of intervention and friendship threshold. Finally, we did not find homophily driven by social norms group, however, there are social network measures associated with homophily like transitivity and reciprocity. Deciding which students should be peer supporters, a subject which has been further investigated in a series of simulation studies^44,49,50^, needs further exploration, particularly concerning the students' attributes and diverse network measures which may help reach and impact the students with social norms more favorable toward smoking and potentially increase the effectiveness of the program.

## Methods

### Study design and participants

We conducted behavioral economic experiments on two data collection time-points (baseline and follow-up) in six schools in NI and six in COL. For the 12 schools, we delivered two different smoking prevention interventions (ASSIST and Dead Cool). Schools were randomly assigned to receive the ASSIST or the Dead Cool intervention, three schools per country and intervention. A total of 1444 students participated in the study, including n = 796 who received the ASSIST intervention (n = 423 in NI; n = 373 in COL), and n = 648 who received the Dead Cool intervention (n = 295 in NI; n = 353 in COL). All students in a whole school year group (target age 12–15 years) were invited to participate (Year 9 in NI and Year 7 in COL). In total, 91.7% of those eligible participated in the study (94.3% in NI and 87.9% in COL). The studies involving human participants were reviewed and approved. Ethical approval has been granted from the Queen’s University Belfast, School of Medicine, Dentistry and Biomedical Sciences Ethics Committee (reference number 18.43; v3 Sept 2018), and by the Ethics committee of the Universidad de Los Andes, Bogotá (937—July 30, 2018). Written informed consent to participate in this study was provided by the participant's legal guardian/next of kin. All research was performed in accordance with relevant guidelines/regulations of Northern Ireland and Colombia. The study has been listed on the ISRCTN registry with trial registration number ISRCTN14041907. It can be viewed at https://www.isrctn.com/ISRCTN14041907.

The ASSIST intervention takes advantage of peer influence. This is based on the Theory of Diffusion of Innovations, including the identification and recruitment of support partners, who, after being trained, are expected to spread prevention messages through their daily and informal relationships^25^. In the short term, it is hypothesized that peer supporters will become more knowledgeable about tobacco and its health consequences, reducing their intentions to engage in tobacco-related behaviors and share tobacco information with their friends. Early adopters are expected to experience a change in knowledge and attitude toward tobacco-related behaviors. In the medium term, due to the increases in social support, self-efficacy to quit tobacco or remain tobacco-free should increase. In addition, the remaining individuals in the social system who are less connected are encouraged to have the same change and acceptance of knowledge due to a higher perception of peer support and modified social norms. In the longer term, the intervention is expected to result in reduced rates of initiation of tobacco-related behaviors^25^. For the ASSIST intervention was conducted a first questionnaire in which all students nominated up to 15 classmates across the 3 questions whom they view as influential (who do you respect/who are good leaders / who do you look up to in your year at your school?). The top 18% of nominated students were invited to a two-day training course where they were trained to be peer supporters and were asked to intervene informally in everyday conversations to encourage their peers to not smoke^26^.

The Dead Cool intervention seeks to impact the classroom pedagogy and is designed to be delivered by teachers, who are trained prior to sessions. The intervention is based on various behavior change techniques such as providing information about consequences, information about the influence of daily relationships, problem-solving and social support^25^. In this intervention, in the short term, it is hypothesized that the information channels of the intervention should lead to greater knowledge about tobacco and its consequences for health. It is also hoped to arouse fear, anticipate regret, and generate greater awareness of available sources of support. In the medium term, it is expected that perceived social support will increase and that changes in perceived social norms should lead participants to form intentions not to smoke. In the longer term, the intervention is expected to reduce rates of initiation of tobacco-related behaviors^25^. For the Dead Cool intervention, teachers were trained to deliver eight weekly lesson plans in their class groups. The lessons examined the influences on smoking behavior from friends, family members, and the media^27^. Since both interventions were developed in the UK, extensive cultural adaptation was undertaken to implement the interventions in COL^53^.

Besides, data were collected before and after the delivery of the interventions using two instruments (more detailed information can be obtained in the published protocol paper^25^). First, a self-administered survey was used to collect data including sociodemographic information, self-efficacy, self-report social norms, and pro-sociality. This survey also included a social network questionnaire asking participants about peer relationships in the same year group, including closest school friends, peers with whom they would talk about something that was upsetting you, peers with whom they spend time outside of school, and influential peers^25^. For this analysis, the closest school friends’ relationship was used (“Please name up to ten of your closest friends in your school year”). This friendship information is independent of the influential actor information used to select the students for the ASSIST intervention. Second, as an experimentally derived measure of social norms, an incentivized experiments questionnaire (based on Game Theory) was conducted. In this experiment, participants were provided with financial incentives to match their answers to other participants in their school year group^28^. The aim of the experiments was to collect a measure of general norms sensitivity, injunctive norms of altruism, injunctive norms about smoking behavior, descriptive norms about smoking behavior, and willingness to pay to support anti-smoking norms^25,42^.

Qualtrics software was used to collect all data (Qualtrics, Provo, Utah, USA Version Jan. 2019). All the instruments were translated and back-translated by bilingual speakers/translators. Questionnaire items are summarized in Supplementary File 1.

### Students attributes

#### Sociodemographic variables

We included the following demographic variables: age, sex, home composition, and ethnic status. Age was categorized as: 11, 12, 13, 14, or 15 or more years old. Sex could be one of the following options: “girl”, “prefer not to say”, and “boy”. Home composition indicated whether the participant lives with a “single parent”, “both parents”, or “other adults”. Ethnic minority status was a binary variable measured by self-identification as part of an ethnic group ("ethnic minority" or "no ethnic minority"). In Northern Ireland, ethnic minorities included "African", "Asian (non-Chinese)", "Chinese", and "any other ethnic group", while non-ethnic minorities included "White Irish" and "White British". In Colombia, ethnic minorities included "Indigenous", "Gypsy/Rom", "Archipelago's Raizal", "San Basilio's Palenquero", "Afro", while non-ethinic minorities included “other ethnic group”.

#### Psychosocial characteristics

We obtained self-report outcomes, including self-efficacy, pro-sociality, and personality traits, from the students in the baseline self-administered survey. Self-efficacy was assessed on a single scale. Pro-sociality was assessed with three variables: Need to Belong Scale, Fear of Negative Evaluation Scale, and Pro-Social Behavior Scale. The personality traits were assessed with the five subscales of the 'Big Five' personality questionnaire: Openness, Extraversion, Agreeableness, Conscientiousness, and Emotional Stability. Further details about the survey and outcomes can be found in the MECHANISMS study protocol^25^.

#### Intervention

For each student, a categorical variable indicating the intervention in which they participated (ASSIST or Dead Cool) was assigned, and a binary variable representing if the student was selected as a peer supporter in the ASSIST intervention was assigned.

#### Social norms measures

Both injunctive and descriptive smoking social norms were measured with a self-report survey and an incentivized experiment with a total of 25 variables^25^. Beliefs about what others do are descriptive social norms, and beliefs about what others think one should do are injunctive social norms^10,11^. This section describes the data treatment to reduce the dimensionality of the indicators describing injunctive and descriptive social norms obtained from the self-report survey and the incentivized experiment. We measured Cronbach's alpha for each construct to assess if the data fit the hypothetical construct as a measure of internal reliability^54^.

In order to reduce the dimensionality of the social norms data, we aggregated the variables obtained from the self-report survey and game theory experiments into the constructs described below (*Self-Report Survey reporting injunctive norms, Self-Report Survey reporting descriptive norms, Incentivized experiment reporting injunctive social norms, and Incentivized experiment reporting descriptive social norms*). To achieve this, first, we standardized and aggregated the variables by dividing in the maximum value to align the sense of the variables in a unique direction, so the value ranged from 0 (against smoking) to 1(favorable towards smoking). With these new variables, we conducted two Confirmatory Factor Analysis (CFA) models, one to obtain a latent injunctive social norm variable, and the other to obtain a latent descriptive social norm variable. A CFA to evaluate the constructs for each time-point. In previous studies of the MECHANISM project by Murray et al^16^ tested whether the data fit the hypothesized measured model for latent variables using a CFA and estimated the loadings of each item used to create the latent variable.

The input variables for both CFAs were the self-report survey indicators and the incentivized experiment indicators standardized by the maximum value and changing the directionality of the metric to align them^16^. The latent injunctive and descriptive norms variables were weighted with the weighted values for the CFA. This resulted in two latent variables (latent descriptive norms and latent injunctive norms), which were the inputs for the following analyses in addition to the sociodemographic variables. The CFA was conducted using the 'lavaan' library on R.

The self-report survey collected information about family injunctive norms, peer injunctive norms, family descriptive norms, and peer descriptive norms^55^. Self-report *injunctive* norms were measured with a seven-point Liker scale, assessing individuals' beliefs about whether important others (e.g. parents, family, and friends) would approve of their smoking. The self-report *descriptive* norm was collected using a six-point Liker scale that assesses the adolescents' perceived smoking behavior of important others (e.g. parents, family, and friends).

The incentivized experiment used coordination games to elicit *injunctive* and *descriptive* social norms for smoking and vaping behaviors. In this experiment, participants were provided with financial incentives to match their answers to other participants' in their school year group^28^. The incentivized *descriptive* norms items measured beliefs about the proportion of the school year group who would be accepting of a close friend smoking or vaping. The incentivized *injunctive* norms assessed beliefs about how most others in the school year group would rate the social appropriateness of eight smoking-related scenarios.

##### Self-report survey reporting injunctive norms

Students answered seven items to assess the perceived norms of the people who are important to them: "most people who are important to me", mother, father, brothers, sisters, friends, and best friend; The survey asked about what the person from the specified relationship thinks and the answering system consisted of a five-point scale from ‘I definitely should smoke’ to ‘I definitely should not smoke’(4, 5), or “I don’t have one” when the participant did not have the relationship (6). To reduce the dimensionality of the obtained data in the self-report survey reporting injunctive norms from seven items to one variable, an average score of the items was calculated for each student. The Cronbach Alpha results of 0.82 and 0.85 for before and after the intervention periods were used as empirical composite reliability measures of the resulting indicator for injunctive norms.

##### Self-report survey reporting descriptive norms

The survey included five items measuring descriptive norms for smoking (e.g. Does your mother smoke?) measured on a six-point Likert scale ranging from “Very often” to “Never”. In addition, three items (e.g. How many of your friends smoke?) measured descriptive norms using a six-point Likert scale ranging from “Almost all of them” to “I have never seen them/I don't know” (6). For both scales, the students could select “I don’t have one” when a relationship did not exist. All the items were grouped in two subscales (family and peer descriptive norms aggregation) using an average score of the existing relationships (4, 7, 8). The family descriptive norms aggregation included mother, father, brothers, sisters, and other family members—Cronbach Alpha coefficients of 0.65 and 0.67 were estimated before and after the interventions. The peer descriptive norms aggregation included friends, best friend, and classmates—Cronbach Alpha coefficients of 0.64 and 0.65 were estimated before and after the interventions. When the student answered not having that relationship, that item was omitted.

##### Incentivized experiment reporting injunctive social norms

The experiment consisted of an incentivized coordination game, where students were provided with financial incentives to match their ratings to other participants' in their school year group, instead of providing personal opinions^28^. Injunctive norms were assessed by asking students to evaluate the social appropriateness of eight smoking-related items using a six-point Likert scale ranging from “Extremely socially inappropriate” to “Extremely socially appropriate”. The answers were aggregated using the mean value in four groups: peer social influence, influence of the tobacco control policies related to the smoke-free environment, influence of the tobacco control policies related to control supply, and influence of the tobacco control policies related to control advertising.

First, peer social influence included in the following situations: (1) an older student from your school smoking outside the school; (2) a school student smoking an e-cigarette; (3) a student sharing a photo of his/her e-cigarette use; and (4) a school pupil chewing tobacco. The Cronbach Alpha coefficients were 0.53 before and 0.65 after the interventions. Second, the influence of the tobacco control policies related to the smoke-free environment included the following situations: (1) a parent smoking at home in front of their young children; and (2) an adult smoking in a car with children on board. The Cronbach Alpha coefficients were 0.76 and 0.79. Third, the influence of tobacco control policies related to control supply included the situation of someone selling cigarettes without proof of age. Finally, the influence of tobacco control policies related to control advertising included the situation of a movie showing the lead character smoking.

##### Incentivized experiment reporting descriptive norms

To assess descriptive norms, the participants were asked to answer two items indicating whether their school peers would be accepting a close friend smoking and vaping using a six-point Likert scale ranging from “none of my peers” to “all of my peers”. They were given financial incentives to match their ratings to other participants' in their school year group, instead of providing personal opinions. The Cronbach Alpha coefficients were 0.84 and 0.85 before and after the interventions. We standardized the resulting variable between 0 (self-report injunctive social norms against smoking) and 1 (self-report injunctive social norms favorable towards smoking) for each student. The construct validity of the incentivized questionnaire was previously tested by Murray et al.^16^.

### Statistical analysis

#### Uncovering the latent groups related to social norms

First, we conducted an initial exploration of how students could be clustered using the defined injunctive and descriptive social norms measures with a Component-based Feature Saliency for Clustering (CFSC). The CFSC identifies clusters in problems with multiple characteristics. Then, to understand how the latent groups changed over time, we conducted a Latent Transition Analysis (LTA). The LTA uncovers the patterns that characterize the clusters of students according to their descriptive and injunctive social norms and estimates how the students transition across the groups over time. Students from both settings with no missing data on the descriptive and injunctive norms items were included (n = 1018).

##### Component-based feature saliency for clustering

We addressed the special characteristics of each sample in distinguishing a cluster rather than focusing on selecting a subset of samples. To do this, we quantitatively measured the clustering relevance of each sample to a cluster, which we refer to as component-based feature saliency. To achieve this, we assumed that the probability distribution of the samples can be modeled as a Gaussian mixture model (GMM). To estimate the feature saliency we used mixture models, and to optimize the clustering results we used Bayesian parameter estimation with Markov Chain Monte Carlo (MCMC) sampling for those cases where no analytical solution was possible^29^.

We here explain how the proposed component-based feature saliency works for mixture model-based clustering. Assume we have a set of N data points y $=\{{{\varvec{y}}}_{1},\dots ,{{\varvec{y}}}_{N}\}$ where each ${{\varvec{y}}}_{i}\upepsilon {\mathbb{R}}^{D}$ is a vector of D features. We use the following probability model for the data distribution:

1 $$p\left({\varvec{y}}|\boldsymbol{\Theta }\right)=\sum_{j=1}^{K}{\alpha }_{j}p({\varvec{y}}|{\boldsymbol{\Theta }}_{j})$$

where ∀j, 0 < ${\boldsymbol{\alpha }}_{{\varvec{j}}}$< 1, $\sum_{j=1}^{K}{\boldsymbol{\alpha }}_{{\varvec{j}}}$=1; each ${\boldsymbol{\Theta }}_{j}$ is the set of parameters of the jth component; $\boldsymbol{\Theta }$= {${\alpha }_{1},\dots ,{\alpha }_{K},{\boldsymbol{\Theta }}_{1},\dots ,{\boldsymbol{\Theta }}_{K}\}$ is the full parameter set. We have a set of missing labels, $Z=\{{{\varvec{z}}}_{1},\dots ,{{\varvec{z}}}_{N}\}$ where ${{\varvec{z}}}_{i}=\{{{\varvec{z}}}_{i1},\dots ,{{\varvec{z}}}_{iK}\}$ with ${{\varvec{z}}}_{ij}=1$ and ${{\varvec{z}}}_{iK}=0$, for k ≠ j, where ${{\varvec{y}}}_{i}$ is a sample of $p({\varvec{y}}|{\boldsymbol{\Theta }}_{j})$. i, j and l index the data sample number, mixture component and feature respectively.

Assume that the features are conditionally independent given the component label, as follows,

2 $$p\left({\varvec{y}}|{\boldsymbol{\Theta }}_{j}\right)=\prod_{l=1}^{D}p({y}_{l}|{\theta }_{jl})$$

where ${y}_{l}$ denotes the lth feature and ${\theta }_{jl}$ is the parameter of the lth feature in the jth component. Combining the above two equations, we have,

3 $$p({\varvec{y}}| \boldsymbol{\Theta })= \sum_{j=1}^{K}{\alpha }_{j}\prod_{l=1}^{D}p({y}_{l}|{\theta }_{jl})$$

To represent the relevance of the lth feature to the jth component of the mixture, we introduce a set of binary parameters $\mathcal{B}=\{{\beta }_{jl}\}$. If the lth feature is relevant to the jth mixture, then ${\beta }_{jl}$ = 1, otherwise ${\beta }_{jl}=0$. The distribution of the lth feature has a common density $p\left(*\right|{\vartheta }_{l}$), where ${\vartheta }_{l}$ is the parameter of the lth feature. The mixture density can be written as:

4 $$p\left({\varvec{y}}\right|\mathcal{B}, \left\{{\alpha }_{j}\right\}, \left\{{\theta }_{jl}\right\},\{{\vartheta }_{l}\})=\sum_{{\varvec{j}}=1}^{{\varvec{K}}}{\alpha }_{j}\prod_{{\varvec{l}}=1}^{{\varvec{D}}}{\left[p\left({y}_{l}|{\theta }_{jl}\right)\right]}^{{\beta }_{jl}}{[q\left({y}_{l}|{\vartheta }_{l}\right)]}^{1-{\beta }_{jl}}$$

We introduce another variable $\mathcal{P}=\{{\rho }_{jl}\}$, ${\rho }_{jl}=P({\beta }_{jl}=1)$, called the component-based feature saliency, the probability that the lth feature is relevant to the jth component. As $P\left({\beta }_{jl}=0\right)=1-{\rho }_{jl}$, we reach:

5 $$P\left({\beta }_{jl}|{\rho }_{jl}\right)= {\rho }_{jl}^{{\beta }_{jl}}{(1-{\rho }_{jl})}^{1-{\beta }_{jl}}$$

Now, we can generate:

6 $$p({\varvec{y}}| \boldsymbol{\Theta })= \sum_{j=1}^{K}{\alpha }_{j}\prod_{l=1}^{D}({\rho }_{jl}p({y}_{l}|{\theta }_{jl})+(1-{\rho }_{jl})q({y}_{l}|{\vartheta }_{l}))$$

where $\boldsymbol{\Theta }$= {$\left\{{\alpha }_{j}\right\}, \left\{{\rho }_{jl}\right\},\left\{{\theta }_{jl}\right\},\{{\vartheta }_{l}\}\}$ is the set of all the parameters of the model.

Let ($\mathcal{Y}, \mathcal{Z}$) be the complete data set. The density of ($\mathcal{Y}, \mathcal{Z}$) is:

7 $$p(\mathcal{Y}, \mathcal{Z}| \boldsymbol{\Theta })=\prod_{{\varvec{i}}=1}^{{\varvec{N}}}\prod_{{\varvec{j}}=1}^{{\varvec{K}}}{[{\alpha }_{j}\prod_{l=1}^{D}({\rho }_{jl}p({y}_{il}|{\theta }_{jl})+(1-{\rho }_{jl})q({y}_{il}|{\vartheta }_{l}))]}^{{\mathcal{Z}}_{{\varvec{i}}{\varvec{j}}}}$$

and $P({z}_{ij}=1$) = ${\alpha }_{j}$, α = (${\alpha }_{1},\dots ,{\alpha }_{K})$ which satisfies

8 $$P\left({{\varvec{z}}}_{{\varvec{i}}}|\alpha \right)=\sum_{j=1}^{K}{({\alpha }_{j})}^{{{\varvec{z}}}_{{\varvec{i}}{\varvec{j}}}}$$

Given ${\theta }_{jl}=({\mu }_{jl},{\sum }_{jl})$ and ${\vartheta }_{l}{\stackrel{-}{=(\mu }}_{jl},{\sum^{-}}_{jl})$, the model likelihood function can be written as:

9 $$p(\mathcal{Y}| \boldsymbol{\Theta })=\prod_{i=1}^{N}\{\sum_{j=1}^{D}{\alpha }_{j}\prod_{l=1}^{D}[{\rho }_{jl}p\left({y}_{il}|{\mu }_{jl},{\sum }_{jl}\right)+(1-{\rho }_{jl})q({y}_{il}|{\overline{\mu }}_{jl},{\sum^{-}}_{jl}]\}$$

The aim of mixture model-based clustering includes: (1) To infer $\boldsymbol{\Theta }$ from the dataset $\mathcal{Y}$,; and (2) to assign each data point to different components, i.e. unveil the unobservable $\mathcal{Z}$.

To determine the parameters of the model shown above, one of the feasible approaches is to deploy Markov Chain Monte Carlo (MCMC) that offers a powerful and flexible method to produce optimal solutions towards the model parameters {$\alpha ,\mathcal{P}, \mu , \sum , \widehat{\mu , } \widehat{\sum })$ for each K. among many MCMC algorithms, the Gibbs sampler is the most commonly used approach in Bayesian mixture estimation. Details of the parameter estimation can be found in ^29^ and we omit this process here.

##### Latent transition analysis

The LTA is a longitudinal extension of Latent Class Analysis (LCA), a multivariate approach that allows the uncovering of hidden grouping variables. This methodology has been extensively studied due to its importance in identifying latent status membership probabilities, and transition probabilities to capture transitions between latent classes over time^56^.

When the LTA modeling exhibits substantive separation in the group membership probabilities of each student, covariates do not have to be included in the estimation of the latent groups. The estimation of the latent groups and the LTA was performed using the MPlus program^57^. To estimate the association of the covariates (sociodemographic and psychosocial traits) with the latent groups at baseline, a multinomial logistic regression was performed using the latent groups from the LTA as the dependent variable and covariates as group predictors^58^. The estimations were performed using the 'nnet' package in R^59^.

The LTA is a type of Latent Markov Model that models longitudinal data for individuals by estimating the incidence of transitions of individuals between latent classes^60^. Assume we have *n* latent classes that are estimated based on longitudinal data that includes *M* variables measured at each time *T*. Let

10 $${Y}_{i}=\left({Y}_{i11},{Y}_{i12},\dots ,{Y}_{i1M},{Y}_{i21},{Y}_{i22},\dots ,{Y}_{i2M},\dots ,{Y}_{iT1},{Y}_{iT2},\dots ,{Y}_{iTM}\right)$$

*Yi* represent the response vector of individual *i*, for each time-point up to *T*, on *M* variables, where responses for individual *i* at time-point *t* on variable *m* is indicated by *Yitm*. These continuous variables are measures at *T* time-points and influence the latent transition probabilities. The injunctive norms and descriptive norms products of the CFA at both time-points are the inputs of the LTA which are used to generate the latent classes at each time-point. The estimation of the latent classes in the second time-point are influenced by the latent classes estimated in the first time-point. The model assumes measurement invariance across time for the latent class variables^30,61^.

#### Assessing the change in network structure over time

Next, we evaluated if there were structural changes over time in the network of friends, undertaking a temporal analysis. For this, we calculated the Jaccard index ${J}_{i}$^62^ that measures the amount of change in the ties of the network over time using the following equation.

11 $${J}_{i}=\frac{{A}_{(\mathrm{1,1})}}{{A}_{(\mathrm{0,1})}+{A}_{(\mathrm{1,1})}+{A}_{(\mathrm{1,0})}}$$

where ${A}_{(\mathrm{1,1})}$ represents the number of ties that existed before and after the intervention, ${A}_{(\mathrm{0,1})}$ represents the number of ties that did not exist before the intervention but existed after the intervention, and ${A}_{(\mathrm{1,0})}$ represents the number of ties that existed before the intervention, but ceased to exist after the intervention. According to the literature, to conclude that there were significant structural changes in the network over time with a gradual change process, values between 0.3 and 0.6 should be obtained^62^.

#### Assessing the relationship between the network structure and the social norms group

To determine the presence of homophily or influence processes, we examined the association between the relationship changes in the structure of the friendship network with the changes in friends’ social norms. Homophily refers to the linkage of students by attributes in common. Influences refer to imitating behaviors of students with which the student is connected. For this, we conducted Separable Temporal Random Graph Models (STERGM) and descriptive analysis.

A STERGM allows us to conclude if there is a greater likelihood of preserving or dissolving ties than that which can be attributed to chance^32,63^. For each network, a STERGM was conducted, including reciprocity and transitivity as network structural variables and the students’ social norms groups as individual attributes. This analysis was conducted using the 'stergm' package in R^32^**.** The STERGMs estimate the likelihood of the formation and dissolution of ties related to social variables over time. This statistical analysis assumes independence between the process of formation and dissolution of ties within the same time-point. Accordingly, a STERGM has two separable formulas. One formula considers the formation process:

12 $$\begin{array}{cc}\mathrm{Pr}\left({Y}^{+}={y}^{+}|{Y}^{t-1}={y}^{t-1};{\theta }^{+}\right)=\frac{\mathrm{exp}\left\{{\eta }^{+}\left({\theta }^{+}\right)*{g}^{+}\left({y}^{+},{y}^{t-1}\right)\right\}}{{c}_{{\eta }^{+},{g}^{+}}\left({\theta }^{+},{y}^{t-1}\right)}& {y}^{+} \in {\Upsilon }^{+}\end{array}\left({y}^{t-1}\right)$$

The other formula considers the dissolution process:

13 $$\begin{array}{cc}\mathrm{Pr}\left({Y}^{-}={y}^{-}|{Y}^{t-1}={y}^{t-1};{\theta }^{-}\right)=\frac{\mathrm{exp}\left\{{\eta }^{-}\left({\theta }^{-}\right)*{g}^{-}\left({y}^{-},{y}^{t-1}\right)\right\}}{{c}_{{\eta }^{-},{g}^{-}}\left({\theta }^{-},{y}^{t-1}\right)}& {y}^{-} \in {\Upsilon }^{+}\end{array}\left({y}^{t-1}\right)$$

with the normalizing constants ${c}_{{\eta }^{+},{g}^{+}}\left({\theta }^{+},{y}^{t-1}\right)$ and ${c}_{{\eta }^{-},{g}^{-}}\left({\theta }^{-},{y}^{t-1}\right)$, the formation statistics ${g}^{+}\left({y}^{+},{y}^{t-1}\right)$, and the dissolution statistics ${g}^{-}\left({y}^{-},{y}^{t-1}\right)$ summing over ${\Upsilon }^{+}\left({y}^{t-1}\right)$ and ${\Upsilon }^{+}\left({y}^{t-1}\right)$ respectively^64^.

With the descriptive analysis, we explored the relationship between the changes in the student's social norm group concerning the change in their friends. Also, we analyzed the friendship threshold according to the proportion of friends (out-degree, i.e. nominations made) that have social norms favorable towards smoking. The analysis was conducted using only the students with complete social norms data at baseline and follow-up.

## Supplementary Information

Supplementary Information 1.

Supplementary Information 2.

## Data Availability

The datasets generated during and/or analyzed during the current study are available from the corresponding author on reasonable request.

## Code availability

The code used to generate the results of the CFA and STERGM reported in the current study is available for replication at https://github.com/mmblanco/Social-network-and-social-norms. The code used to generate the results of the CFSC has been cited and is available at https://github.com/XHongs/Component-based-Feature-Saliency-for-Clustering. The code used to generate the results of the LTA (MPlus software) is available from the corresponding author on reasonable request.
