# Supplementary material for: Exploring the mechanistic pathways of how social network influences social norms in adolescent smoking prevention interventions
Source: Sci Rep. 2023 Feb 21;13:3017. doi: 10.1038/s41598-023-28161-7 (PMC9944961; doi:10.1038/s41598-023-28161-7)
Supplement: Supplementary file 2 — Supplementary Information 2. [file 41598_2023_28161_MOESM2_ESM.docx]

**Supplementary Information for**

Exploring the mechanistic pathways of how social network influences social norms in adolescent smoking prevention interventions.

Felipe Montes^1^, Martha Blanco^1^, Andres Felipe Useche^1^, Sharon Sanchez-Franco^2^, Carlos Caro^1^, Lei Tong^3^, Jie Li^4^, Huiyu Zhou^3^, Jennifer M. Murray^5^, Olga L. Sarmiento^2^, Frank Kee^6^, Ruth F. Hunter^6^.

1. Department of Industrial Engineering, Social and Health Complexity Center, Universidad de Los Andes, Bogotá, Colombia
2. Department of Public Health, School of Medicine, Universidad de Los Andes, Bogotá, Colombia
3. School of Informatics, University of Leicester, Leicester, United Kingdom
4. College of Electronic and Information Engineering, Nanjing University of Aeronautics and Astronautics, China.
5. Centre for Public Health, Institute of Clinical Sciences, Block B, Queen's University Belfast, Royal Victoria Hospital, Grosvenor Road, Belfast, BT12 6BA, 02890978955, Queen's University Belfast, Belfast, United Kingdom
6. Centre for Public Health, Institute of Health Sciences, School of Medicine, Dentistry and Biomedical Sciences, Queen’s University Belfast, Belfast, United Kingdom, BT12 6BJ.

Felipe Montes

Email: fel-mont@uniandes.edu.co

**This PDF file includes:**

Supplementary text for Results.

Table S1

SI References

Supplementary Information Text

# RESULTS

# Uncovering the groups related to social norms

### Reducing the dimensionality of the data

Using a Confirmatory Factor Analysis (CFA), we reduced the 25 social norms indicators into two factors: injunctive norms and descriptive norms. The Cronbach Alpha coefficients and the results from the CFA concluded that the data fit the hypothetical construct explained in section 1.1 of the SI Appendix.

Table S1 shows the results from the CFA (n=1620). To determine model-fit, we extracted a number of fit indices, and obtained values of 96.9% for the Comparative fit index, 94.7% for the TuckerLewis Index, 0.03 for the Root Mean Square Error of Approximation (RMSEA), and a p-value of less than 0.001 for the chi-square test statistic. This demonstrated that the model fit the data well(1). The resulting two latent variables were used in the analysis from the main text.

### Social norm groups: initial classification with a Component-based Feature Saliency for Clustering

We identified latent groups and their changes over time, between baseline and follow-up after the smoking prevention interventions, for the smoking-related norms outcomes in the schools. These groups were classified according to *descriptive* and *injunctive* social norms favorable towards smoking or against smoking. We conducted an initial classification with a Component-based Feature Saliency for Clustering (CFSC) (12). From the initial exploration with the CFSC approach, we obtained two groups (clusters) at baseline (N_1_=772 and N_2_=246) and three at the follow-up (N_1_=590, N_2_=155, and N_3_=273). At baseline, group 1 was characterized by students with *descriptive* and *injunctive* social norms against smoking, and group 2 was characterized by students with *injunctive* social norms more favorable towards smoking irrespective of their perceived *descriptive* norms. At follow-up, group 1 and group 3 were characterized by students with *descriptive* and *injunctive* social norms against smoking while group 2 continued to represent students with *injunctive* social norms more favorable towards smoking irrespective of their *descriptive* norms. From baseline to follow-up, the number of students in the group with *injunctive* social norms favorable towards smoking (group 2) decreased (from 246 to 155).

### Incentivized and self-report social norms

We did a second type of aggregation of the variables to assess differences when grouping the incentivized and self-report social norms. We conducted a CFA to obtain a (1) self-report *injunctive* norm, (2) experimentally derived *injunctive* norm, (3) self-report *descriptive* norm, and (4) experimentally derived *descriptive* norm. The experimentally derived *descriptive* norms and the self-report norms corresponds to the average of their indicators. The experimentally derived *injunctive* norms and the self-report *descriptive* norms were estimated using two CFAs with the constructs defined in section 1.1. Good model fit was determined with a Comparative fit index of 96.6%, Tucker-Lewis index of 89.7%, RMSEA of 0.09 and the chi-square statistic p-value of 0.003.

Then, we conducted two Latent Transition Analyses (LTA), the first with the self-report norms and the second with the experimentally incentivized norms. The LTA identified two groups for both the experimentally derived norms and the self-report norms. Regarding the experimentally derived norms, group 1 (N=821 at baseline; N=850 at follow-up) was characterized by students with *descriptive* social norms against smoking regardless of their *injunctive* social norms, while group 2 (N=197 at baseline; N=168 at follow-up) was characterized by students with *descriptive* social norms more favorable towards smoking, regardless of their *injunctive* social norms. Regarding the self-report norms, groups definitions differed over time. At baseline, group 1 (N=914) was characterized by students with *descriptive* social norms against smoking regardless of their *injunctive* social norms, and group 2 (N=104) was characterized by students with *descriptive* social norms more favorable towards smoking regardless of their *injunctive* social norms. However, at follow-up, group 1 (N=97) was characterized by students with *injunctive* social norms more favorable towards smoking regardless their *descriptive* social norms, and group 2 (N=921) was characterized by students with *injunctive* social norms against smoking, regardless of their *descriptive* social norms.

We observed that group definitions were mainly given by the *descriptive* social norms (i.e. observable variables), while the *injunctive* social norms (i.e. latent variables which are not directly observable) were relevant only in one from the four possible group definitions. Also, the groups’ transitions (changes) for the experimentally derived norms, for which the group definitions were maintained overtime, behaved in a similar way to the groups analyzed in the main text.

**Table S1.** Confirmatory Factor Analysis construct validation

|  | **INJUNCTIVE** | | **DESCRIPTIVE** | |
| --- | --- | --- | --- | --- |
| **CFA** | **T0** | **T1** | **T0** | **T1** |
| **Fit Index** | 97% | 95% | 100% | 100% |
| **Tucker-Lewis Index** | 93% | 91% | 100% | 100% |
| **RMSEA** | 0.065 | 0.091 | <0.001 | <0.001 |
| **p-value** | <0.001 | <0.001 | <0.001 | <0.001 |

# SI References

1. D. Hooper, J. P. Coughlan, M. R. Mullen, Structural equation modelling: guidelines for determining model fit. *Electron. J. Bus. Res. Methods*, 53–60 (2008).
